# Shoes size can predict implant sizes for primary total knee arthroplasty in a quick, reliable and costless manner

**DOI:** 10.1002/jeo2.70363

**Published:** 2025-07-18

**Authors:** Corentin Philippe, Alexandre Le Guen, Nicolas Vari, Pablo Froidefond, Gary Kolenc, Emilie Berard, Etienne Cavaignac

**Affiliations:** ^1^ Institut Loco‐Moteur, Hôpital Pierre Paul Riquet, CHU Toulouse Toulouse France; ^2^ Société Antipodes Toulouse France; ^3^ Service d'Epidémiologie, CHU Toulouse, CERPOP, Inserm, Université Toulouse III Paul Sabatier Toulouse France; ^4^ I2R, Institut de Recherche Riquet Toulouse

**Keywords:** component size, preoperative planning, shoe size, total knee arthroplasty

## Abstract

**Purpose:**

Knowing the component sizes needed for a specific patient before total knee arthroplasty (TKA) surgery could help to optimise the logistics of medical device availability. Previous studies have correlated component size with patient age, sex, height, weight, and shoe size, but none have validated this method using the European shoe sizing system. The primary objective of this study was to determine the correlation between a patient's European shoe size at the time of surgery and the size of the tibial and femoral components used during primary TKA. The secondary objective was to evaluate the accuracy within ±1 size between the European shoe size and the component size.

**Methods:**

This was a retrospective observational, single‐centre, single‐surgeon study of 227 primary TKA procedures done with the Score II implant (AMPLITUDE®, Valence, France) between 1 April 2022 and 1 July 2023. Data on the patient's shoe size was determined before the surgery. This information was retrospectively correlated with the size of the components used in the TKA surgery that was recorded in the operative report.

**Results:**

The correlation between a patient's shoe size and the component size was very strong: Spearman rho of 0.8095 for femur, 0.8400 for tibia and 0.6393 for patella (*p* < 0.001). The correlation between a patient's shoe size and the size of the PE insert was weak: Spearman rho 0.1532 (*p* = 0.0210). After adjusting for sex, the femoral component was predicted accurately within ±1 size in 92% (210/227) of procedures and the tibial component in 94% (213/227). After adjusting for sex and BMI, the patellar implant was predicted accurately within ±1 size in 97% (220/227) of procedures.

**Conclusion:**

Our model provides a cost‐effective, assignable and easily implementable method for predicting tibial and femoral component sizes using European shoe size, demonstrating high accuracy (≥92%).

**Level of Evidence:**

Level IV, retrospective observational study.

**Abbreviations**
OR: operating room
PE: polyethylene
TKA: total knee arthroplasty
UKA: unicompartimental knee arthroplasty

## INTRODUCTION

The number of total knee arthroplasty (TKA) procedures done annually continues to increase, some of which are done as same‐day procedures, despite worldwide supply issues affecting medical device availability [2, 10]. Knowing the component sizes appropriate for a patient before the TKA surgery could help to optimise the logistics. This is even more important in a healthcare facility that has a high volume of arthroplasty procedures or conversely, in a facility that rarely does this surgery. It is no longer necessary to always have a large stock of implants with multiple units of every size [2]. This reduction in stock could reduce costs by reducing the area in the operating room (OR) suite dedicated to storage, while also reducing the time spent by the nurse who needs to regularly check the expiry date of these implants. And knowing in advance the component size would save operating time since the circulating nurse would already have the component in the OR, instead of having to leave the OR to get it from the supply room. For facilities where an entire shelving unit with all the component sizes is brought to the OR, this would eliminate the need for a nurse to handle heavy loads.

Preoperative planning is not widely done for TKA because the procedure's instrumentation is very precise, making the intraoperative selection of components easy and accurate. Preoperative templating methods on radiographs are time‐consuming, difficult for a surgeon to delegate and require standardised radiographs [6, 8]. Also, the software can be costly [11].

Previous publications have described how to predict component sizes based on a patient's characteristics. In the unicompartmental knee arthroplasty (UKA) study by Sawalha et al., the shoe size could predict the size of the femoral component accurately in 80% of cases and was within 1 size in 100% of cases [12]. The study of 498 primary TKA cases by Van Egmond et al. reported accurate prediction of component sizes within 1 size based on shoe size in 94% of femurs and 86% of tibias [3]. Lastly, the Naylor team described a model combining shoe size, height, weight, age and gender that predicted the size ±1 in 83% of the implants needed for a primary TKA [9].

However, these studies were done in English‐speaking countries and did not use the European shoe sizing system, nor did they study all of the available implant models. Thus, a validation study is needed that incorporates the European shoe sizing system and the TKA implant system used at our facility. For this correlation to be used in current practice, we need to develop a chart that links shoe sizes and component sizes for each type of TKA implant.

The primary objective of this study was to determine the correlation between a patient's shoe size at the time of surgery and the size of the tibial and femoral components used during primary TKA. The secondary objective was to evaluate the accuracy within ±1 size between the shoe size and component size used during a primary TKA procedure. We hypothesised that the patients' shoe size at the time of surgery and the size of the femoral and tibial components used during primary TKA are correlated.

## MATERIALS AND METHODS

This was a retrospective observational single‐centre study (RnIPH 2021‐64) that retrospectively analysed data collected prospectively in patients who were being treated with standard of care. It was done at the university hospital of Toulouse by a single surgeon and using a single TKA implant model. This design minimise variability in surgical technique and implant selection. However, this limits the generalisability of findings to different surgical practices and implant brands. Shoe size data was recorded (self‐reported by patient) preoperatively by clinical staff who were blinded to the final implant size selection to prevent measurement bias. The surgeon chose the surgical approach (transquadriceps or subvastus) and the size of the polyethylene (PE) insert. The inclusion criteria were: (1) primary TKA procedure, (2) Score II implant (AMPLITUDE®, Valence, France) and (3) operated between 1 April 2022 and 1 July 2023. The exclusion criteria were: (1) revision TKA procedure, (2) prior UKA, (3) shoe size unavailable at the time of surgery and (4) patient refusing to participate. Cases with missing data were not included in the final analysis The primary outcome was the correlation between the tibial and femoral component sizes and the shoe size at the time of surgery. The secondary outcome was the accuracy within ±1 size of the tibial and femoral component size predicted by the shoe size.

Appendix 1 and 2 give the measurements in millimetres of the components used and equivalences between European, English and American shoes size systems.

To reveal a correlation of 80% with a bilateral confidence interval of 95% with 10% precision, 205 patients without missing shoe size and component size data were needed. The sample size was determined based on prior literature suggesting an 80% correlation between morphological parameters and implant sizes in knee arthroplasty [1]. Thus, we planned to include 250 patients to account for potential missing data. The analysis of a correlation between component size (femoral, tibial, patellar and PE insert) and the patient's shoe size involved calculating the Pearson or Spearman correlation coefficient (depending on their application criteria). Distribution was analysed by the Shapiro‐Wilk test. To calculate the accuracy within ±1 size of the component size predicted by shoe size, a linear regression line was constructed to predict the size of each component as a function of shoe size. The difference between the predicted size and the implanted size was calculated along with the accuracy within ±1 size. The statistical analysis was done with STATA software (version 18.0, STATA Corp., College Station, TX, USA), with a two‐tailed threshold of 0.05.

## RESULTS

The study included 227 consecutive patients; all were analysed. Descriptive data for the study population are shown in Table 1.

**Table 1:** Characteristics of the study population.

Variable	Statistics (*n* = 227)	
Age at surgery (years)	Mean (SD)	67.8 (9.7)
	Min; Max	32; 94
Sex		
Female	*n* (%)	117 (52)
Operated side		
Right	*n* (%)	124 (55)
BMI (kg/m²)	Mean (SD)	28.0 (4.3)
	Min; Max	16.5; 38.7
European shoe size		
	Mean (SD)	41 (3)
	Min; Max	36; 49
Tibial component size		
	Mean (SD)	4 (2)
	Min; Max	1; 8
Femoral component size		
	Mean (SD)	5 (2)
	Min; Max	2; 8
Patellar component size		
	Mean (SD)	34 (3)
	Min; Max	26; 39
PE insert size		
	Mean (SD)	11 (2)
	Min; Max	9; 20

Abbreviations: BMI, body mass index; PE, polyethylene; SD, standard deviation.

The correlation between shoe size and component size was strongest for the tibia (Spearman rho = 0.8400, *p* < 0.001) and femur (rho = 0.8095, *p* < 0.001), with a moderate correlation for the patella (rho = 0.6393, *p* < 0.001). The correlation between the patient's shoe size and the size of the PE insert was weak: Spearman rho 0.1532 (*p* = 0.0210).

The femoral component was accurately predicted within ±1 size in 87% (198/227) of patients, 92% (210/227) after adjusting for sex. The tibial component was accurately predicted within ±1 size in 92% (208/227) of patients, 94% (213/227) after adjusting for sex. There was no statistical correlation between BMI and the size of the tibial or femoral components. The patellar component was accurately predicted within ±1 size in 58% (131/227) of patients, 97% (220/227) after adjusting for sex and BMI.

Tables 2, 3, 4 show the relationship between shoe size and component size in chart format.

**Table 2:** Chart showing the predicted femoral component size relative to shoe size and sex.

Shoe size	Femoral component size						
	Female			Male			
36	3						
37	3						
38		4					
39		4		5			
40		4		5			
41			5		6		
42			5		6		
43			5		6		
44					6		
45						7	
46						7	
47						7	
48							8
49							8

**Table 3:** Chart showing the predicted tibial component size relative to shoe size and sex.

Shoe size	Tibial component size						
	Female			Male			
36	2						
37	2						
38		3					
39		3		4			
40		3		4			
41			4		5		
42			4		5		
43			4		5		
44						6	
45						6	
46						6	
47							7
48							7
49							7

**Table 4:** Chart showing the predicted patellar component size relative to shoe size, sex and BMI.

Shoe size	Patellar component size						
	Female (BMI < 25//BMI > 25)			Male (BMI < 25//BMI > 25)			
36	30//**30**						
37		33//**30**					
38		33//**30**					
39			33//**33**	33//**33**			
40			33//**33**	33//**33**			
41			33//**33**	33//**33**			
42			33//**33**		36//**33**		
43			33//**33**			36//**36**	
44						36//**36**	
45						36//**36**	
46						36//**36**	
47						36//**36**	
48							39//**36**
49							39//**36**

## DISCUSSION

There is a strong correlation (defined as Rho > 0.50) between a patient's European shoe size at the time of surgery and the size of femoral, tibial and patellar components of TKA. Our model based on shoe size and sex was able to accurately predict within 1 size more than 92% of the tibial and femoral component sizes needed intraoperatively.

Bhowmik‐stoker et al. found that weight, height and sex are the primary factors correlated with TKA component size. The component size was accurately predicted within 1 size for 94% of femurs and 96% of tibias [1]. Sheron et al. developed a model based on age, height and weight for predicting the tibial and femoral component sizes of several types of TKA implants. Their prediction was accurate within 1 size for the femoral and tibial component in 71%–92% and 81%–97%, respectively, using morphological data only and 85%–99% and 90%–99% when adding radiological templating and age [13]. Sawalha et al. reports 100% accurate prediction of femoral component size for UKA based on shoe size [12]. Thus, our findings are consistent with these publications since the predicted accuracy was similar to the models based on morphological criteria.

We choose to develop an implant size predicting model based on few data to have a really fast and simple model in daily use. We choose shoes size and sex because they are easily self‐reported by the patient.

The published results for predicting TKA component sizes by radiographic templating are also similar to the results of our models. Levine et al. carried out digital templating of 269 consecutive primary arthroplasty cases and reported accuracy within 1 size for 93% of total hip arthroplasty and 98.5% of TKA cases [8]. Hernandez‐Vaquero et al. carried out radiographic templating at the femur and tibia and reported the exact size was predicted in 55% and 50% of cases, and the size within ±1 in 90% and 94%. The intra‐rater and inter‐rater reliability were good [4]. Lastly, Trickett et al. reported that more than 95% of femoral and tibial component sizes were predicted within ±1 size using radiographic templating.

Thus, the accuracy of predicted TKA implant size with our model is equal to that of published models. However, our model is more efficient as only one parameter (shoe size) needs to be collected, in contrast to existing models that requires height and weight at a minimum, and sometimes standardised radiographs. It is even more efficient in that the component sizing is found instantaneously by reading a chart, does not add extra cost and can be delegated to other members of the healthcare team. Lastly, it does not require additional preoperative work‐up such as a specific imaging protocol, contrary to image‐based templating [4]. These standardised images are a problem for facilities that have a low volume of TKA and/or in medically underserviced areas that cannot always implement the required protocols. Our approach with a simple clinical model is to make the overall process fluid. even if analysis by x‐ray or CT scan is possible, it has an additional cost, whether in terms of the emanation itself (CT scan) or the medical time required for analysis. we are in a context of maximum optimisation with this approach. It is a simple process that can be carried out by the nurse and that will optimise his or her analysis of the case. Lastly, our model has an excellent prediction rate for an implant model that has nine component sizes. Previous studies have shown that the best accuracy for predicting sizes was achieved with implant models that have few sizes [13, 14].

There are many benefits associated with knowing the TKA component sizes needed before the surgery. It helps to optimise the logistics of the OR suite, since the circulating nurse only needs to bring the likely insert sizes to the OR. This helps to reduce the handling and number of trips to the supply room. This also reduces the time between surgery blocks when a non‐arthroplasty surgery and a TKA surgery are scheduled in the same OR because one does not need to bring all the component sizes into the room. Reducing repetitive tasks and material handling may optimise workflow efficiency and reduce paramedical staff fatigue, a critical factor given current global healthcare workforce shortages.

Knowing in advance the implants needed also helps to reduce the permanent stock of implants in the supply room. The facility can order from the manufacturer in advance the likely sizes for patients scheduled in the upcoming weeks and keep only a limited stock of all sizes to cover the rare instances (less than 8%) where the model does not predict the component sizes. Also, the preoperative prediction is essential for patients who need extreme component sizes that must be ordered from the manufacturer before surgery.

Lastly, a priori knowledge of likely component sizes makes the surgeon's life easier. This facilitates the surgeon's intraoperative workload and knowing as priori the size to implant—or to get close—helps to save time by being less progressive in the bone cuts [5, 14]. Knowing the component sizes beforehand also helps to reduce the surgeons' learning curve [5] and provides legal proof of the surgeon's preparation before the surgical procedure [14]. This could also improve the clinical outcomes of TKA, because planning helps to eliminate large errors in component sizes (outliers) that lead to functional failures [4].

Our study has certain limitations. The accuracy of our model is high and useful in daily practice by accepting a one‐size variation in the needed dimension. This implies that a safety stock of all implant sizes is still necessary. However, this stock will be smaller than if the component sizes was only determined intraoperatively. While foot length remains relatively stable throughout life, variations in foot width due to aging, surgery, or oedema may introduce bias in shoe size‐based predictions, particularly in older patients [7, 12]. However, these variations in shoe size do not appear to have a significant effect. Sawalha et al. found no changes in their model's accuracy for predicting UKA component sizes when patients maintained or changed their shoe size during their life [12]. World wide application of our model is limited to only one prosthetic model and need to be validated with other TKA brand and models. Lastly, our model could not accurately predict the thickness of the PE insert for TKA. Van Egmond et al. also found no correlation between shoe size and PE insert thickness [3].

This model has been internally validated but lacks external validation in diverse patient populations and surgical settings. Future studies should replicate these findings across different centres and implant brands.

## CONCLUSION

Our model to predict the femoral and tibial component sizes is simple, low‐cost, reproducible, efficient and delegable. By simply knowing the patient's shoe size and sex, it can accurately predict within ±1 size the femoral and tibial components needed intraoperatively in more than 92% of cases. Our study can be extended by measuring the amount of improvement in OR efficiency and developing a similar model for other TKA implant systems.

## AUTHOR CONTRIBUTIONS

Corentin Philippe: Data analysis and writing of the manuscript. Alexandre Le Guen; Nicolas Vari, Pablo Froidefond, and Gary Kolenc: Data collection. Emilie Berard: Statistical analysis. Etienne Cavaignac: Original idea, data analysis and supervision of the study.

## CONFLICT OF INTEREST STATEMENT

Etienne Cavaignac is paid consultant for Arthrex and Amplitude. Gary Kolenc is paid consultant for Amplitude. Other authors declare that they have no conflict of interest. The other authors declare no conflict of interest.

## ETHICS STATEMENT

This work was a retrospective observational single‐centre study (RnIPH 2021‐64) that retrospectively analysed data collected prospectively in patients who were being treated with standard of care.

## Data Availability

The data sets used and/or analysed during the current study are available from the corresponding author on reasonable request.

## APPENDIX 1: Measurements in millimetres of femoral, tibia and patellar components for each size

**Table:**

Femur	0	1	2	3	4	5	6	7	8
AnteroPosterior	44.6	47.1	49.7	52.3	54.9	57.5	60.1	62.6	65.3
MedioLateral	56	58.1	60.5	63.1	66	69.1	72.4	76	80

**Table:**

Tibia	0	1	2	3	4	5	6	7	8
AnteroPosterior	39.2	41.4	43.6	45.9	48.2	50.5	52.8	55	57.2
MedioLateral	60	63.5	67	70.5	74	77.5	81	84.5	88

**Table:**

Patella	30	33	36	39
Diameter	30	33	36	39
Thickness	8	8	8	8

## 1 APPENDIX 2: Shoes size equivalence between European, English and American systems

**(female (F); male (M))**

**Table:**

Europe	United Kingdom	United States of America
36	3.5	F: 5
37	4	F: 5.5
38	5	F: 6.5
39	5.5	F: 7.5; M: 6
40	6.5	F: 8; M: 7
41	7.5	F: 9; M: 8
42	8	F: 9.5; M: 8.5
43	9	F: 10.5; M: 9.5
44	9.5	M: 10
45	10.5	M: 11
46	11	M: 11.5
47	12	M: 12.5
48	13	M: 13.5
49	13.5	M: 14.5
